# 

**Published:** 1907-07

**Authors:** 


Vol. XXIII. JULY, 1907. No. 1

### TEXAS MEDICAL JOURNAL

"RED BACK"

PUBLISHED AT AUSTIN, TEXAS, BY
F. E. DANIEL, M. D., - - Proprietor
PRINTED BY THE VON BOECKMANN-JONES CO.

Subscription, $1.00 a Year, in Advance. - - - Single Copies, 10 Cents

Entered at the Postoffice at Austin, Texas, as Second-class Mail Matter

